# One-step electrodeposition of ZnO/graphene composites with enhanced capability for photocatalytic degradation of organic dyes

**DOI:** 10.3389/fchem.2022.1061129

**Published:** 2022-11-02

**Authors:** Hui Lu, Simiao Sha, Tong Li, Qian Wen, Shaolin Yang, Jiandong Wu, Kang Wang, Zhilin Sheng, Jinfu Ma

**Affiliations:** ^1^ School of Materials Science and Engineering, North Minzu University, Yinchuan, China; ^2^ Yinchuan Aini Industrial Technology Development Co., Ltd, Yinchuan, China

**Keywords:** ZnO, graphene oxide, electrodeposition, electrolyte, solid catalyst

## Abstract

Zinc oxide is a popular semiconductor used in catalysts due to its wide bandgap and high exciton binding energy. However, the photocatalytic performance of ZnO was compromised by its insufficient electron-hole separation efficiency and electron transfer rate. Herein, ZnO-reduced graphene oxide (rGO) composite solid catalyst was synthesized by one-step electrodeposition method on FTO substrate using lithium perchlorate (LiClO_4_) as the supporting electrolyte. Scanning electron microscopy, Raman, Fourier Transform Infrared, and XRD characterizations confirmed the deposition of ZnO and the reduction of graphene oxide Owing to the cooperative effect between rGO and ZnO, the as-prepared ZnO-rGO composites show much enhanced photocatalytic degradation ability compared with pure ZnO nanorods. By optimizing the conditions of electrodeposition of ZnO-rGO composites, the degradation rate of methylene blue can reach 99.1% within 120 min. Thus, the simple preparation and the excellent performance could endow the ZnO-rGO composites with promising application in practical dye-polluted water treatment.

## Introduction

The growing water contamination has become a serious problem with the development of industrialization and urbanization (Liras et al., 2019; Abdel-Karim et al., 2021). As a kind of staining materials, dyes are widely used in pollution-intensive industries such as textile, rubber, papermaking, plastics, and printing (Mansor et al., 2020; Nasir et al., 2021). Untreated dye-containing industrial wastewater produces severe pollution and threatens the ecological environment and human health. In addition, the organic dye pollutants in the wastewater are hard to be decomposed naturally. Among various dye-removal techniques, the degradation of organic dyes using highly efficient photocatalysts has become the most promising due to its high efficiency, fast reaction, and easy operation (Garg et al., 2020).

Until now, many low-priced, high-performance, and consistent photocatalysts have been produced and used in wastewater treatment, such as, metal oxides (TiO_2_ and ZnO) (Roshni and Thambidurai, 2022; Umadevi et al., 2022), sulfides (Luan et al., 2020; Ren et al., 2021), and nitrogen oxides (Niu et al., 2012). Among them, ZnO, an n-type semiconductor material of the group II-VI has been widely used in recent years because of its wider bandgap and higher exciton binding energy (Dehghan Nayeri et al., 2013). However, the deficiencies of ZnO including high resistivity, and easy recombination of photogenerated electron-hole pairs seriously hinders the improvement of its photochemical catalysis performance (Anandan et al., 2010). To overcome these shortcomings and improve the photochemical catalysis performance, the ZnO composite materials have been widely investigated (Lonkar et al., 2018). As a two-dimensional carbon nanomaterial with zero bandgap, graphene has the advantages of high electron mobility and excellent conductivity at room temperature, making it a candidate for enhancing the performance of various catalysts (Imran et al., 2021; Kharatzadeh et al., 2021; Moradi et al., 2021). For these reasons, ZnO-rGO composite photocatalysts with better optical and electrical properties were synthesized. The addition of rGO can promote the separation of electron-hole pairs and reduce the recombination rate, and increase the light absorption capacity (Abdelsamad et al., 2018). Therefore, the ZnO-rGO composite can obtain better photocatalytic degradation performance than ZnO. Until now, various techniques for synthesizing ZnO-rGO composites have been exploited. For instance, Azaranga et al. fabricated nanocomposites of ZnO NPs and rGO by sol-gel method, and the ZnO-rGO nanocomposites achieved a degradation efficiency of about 92.5% for methylene blue (MB) within 120 min (Azarang et al., 2015). Tuan et al. prepared ZnO/rGO nanocomposites by hydrothermal method, which can only degrade 60% MB in 60 min (Van Tuan et al., 2020). However, most methods suffer from complex preparation process, high temperature and pressure conditions, difficult recycling and other problems, which limits their practical application.

In this paper, we developed a simple, low-cost, controllable method to synthesize ZnO-rGO composite by one-step electrochemical deposition using GO, zinc nitrate (Zn(NO_3_)_2_) and lithium perchlorate (LiClO_4_) solution as electrolyte. The use of LiClO_4_ as supporting electrolyte is conducive to the growth of ZnO and the uniform coverage of rGO nanosheets onto the surface of ZnO nanorods. Since the deposition of ZnO and the reduction of GO were carried out concurrently, the removal of GO with toxic reductants was avoided. Due to the combined effect between rGO and ZnO, the photochemical catalytic activity of ZnO-rGO composites was significantly improved compared with that of pure ZnO nanorods. After optimizing the GO concentration in electrolyte, the photocatalytic degradation rate of MB by ZnO-rGO composites reached 99.1% within 120 min.

## Experimental

### Materials

FTO coated glass (13 ± 1.5 ohm) was purchased from Dalian Qiseguang Solar Technology Development Co., Ltd. Zinc nitrate (Zn(NO_3_)_2_, AR), potassium chloride (KCl, AR) and lithium perchlorate (LiClO_4_, AR) were purchased from Shanghai Aladdin Biochemical Technology Co., Ltd. The graphene oxide (GO) aqueous solution was provided by Suzhou Carbon Fung Technology Co., Ltd.

### Electrodeposition of ZnO-rGO composite materials

All electrodeposition processes were implemented on a CHI660E electrochemical workstation (Chenhua Instruments, China) using three-electrode system comprised of FTO, Pt wire, and Ag/AgCl as the working electrode, counter electrode, and reference electrode, respectively. The FTO conductive glasses were cleaned with ultrasonic oscillation with the glass cleaning agent, deionized water, and ethanol for 30 min successively. ZnO and ZnO-rGO films were electrochemical deposited on FTO substrates by potentiostatic method at 80 °C with electrodeposition potentials and time of −1.1 V and 600 s, respectively. The electrolyte for ZnO nanomaterials was 10 mM Zn(NO_3_)_2_ and 0.1 M LiClO_4_ aqueous solution, whereas the ZnO-rGO was deposited with an electrolyte containing 10 mM Zn(NO_3_)_2_, 0.1 M LiClO_4_, and 5 mg L^−1^ GO. For comparison, ZnO-rGO composite prepared without LiClO_4_ as supporting electrolytes was named as ZnO-rGO-N. To study the effect of GO concentration on the photochemical degradation of the synthesized ZnO-rGO, the ZnO-rGO composites were prepared with GO concentration of 2, 5, and 8 mg L^−1^, which were named ZnO-2rGO, ZnO-5rGO, and ZnO-8rGO, respectively.

### Characterization

Scanning electron microscopy (SEM) and energy dispersion spectroscopy (EDS) mapping were carried out on a Zeiss Sigma 600 field emission scanning electron microscope. X-ray diffraction (XRD) test was conducted on a Rigaku Dmax-2500. X-ray photoelectron spectroscopy (XPS) was performed on an ESCALAB Xi + X-ray photoelectron spectrometer. The ultraviolet-visible spectra were measured by using an ultraviolet-visible (UV-vis) spectrophotometer (Beijing Puxi TU-1901). Raman spectra were measured by HORIBA LabRAM micro-Raman microscope irradiated with a 514 nm laser. Fourier Transform Infrared (FTIR) spectra were recorded by Thermo Scientific Nicolet iS5 FTIR Spectrometer.

### Photocatalytic tests

Organic dyes including MB, Rhodamine B (RhB), and Methylene orange (MO) were used as organic pollutants to measure photocatalytic activity, respectively. Three pieces of FTO (1.5 cm × 1.5 cm) coated with ZnO or ZnO-rGO composite materials were immersed into 30 ml organic dyestuff solution with a concentration of 10 mg L^−1^. To achieve the balance of adsorption/desorption, the solution was placed in the dark for 30 min before measurement. Afterwards, the dyestuff solution was irradiated with a mercury lamp (300 W) from a distance of 10 cm for photodegradation. 3 ml of the dye solution was taken to test its absorbance with an UV-vis spectrophotometer every 20 min and returned to the solution after the test. The degradation efficiency was calculated according to the variation of the maximum characteristic absorption peaks. Transient photocurrent tests were performed in a KCl electrolyte with a bias voltage of 0.5 V and 0.1 mol L^−1^1 mmol L^−1^ of BQ, 10 mmol L^−1^ of IPA and 10 mmol L^−1^ of EDTA-2Na were used as trapping agents for O_2_^−^, ·OH and *h* ^+^, respectively, and the reaction mechanism was tested according to the steps of photocatalytic degradation to analyze the reaction mechanism.

## Results and discussion

**FIGURE 1:**
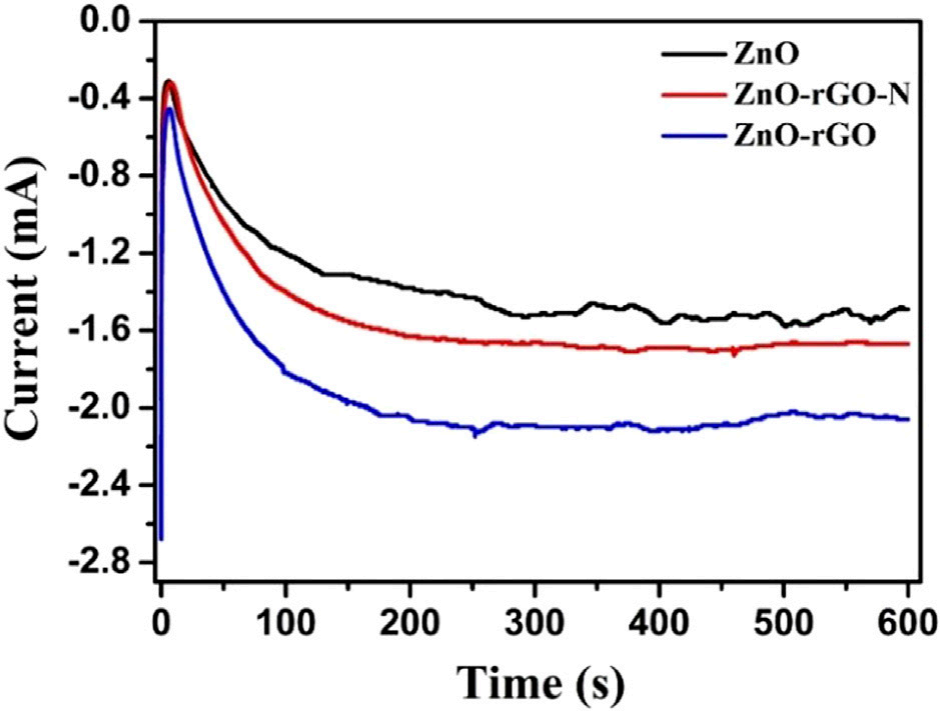
CA curves for the electrodeposition of ZnO, ZnO-rGO-N, and ZnO-rGO at a potential of -1.1 V.

In the process of preparing ZnO-rGO composite materials by one-step electrochemical deposition, the choice of supporting electrolytes determines the quality of the deposited film, which is the key to the photocatalytic performance. Figure 1 shows the Chronoamperometry (CA) curves for electrochemical deposition of ZnO and ZnO-rGO nanocomposites using potentiostatic method. These curves have a similar trend: The rapid decrease of current at the initial deposition ascribed to the rapid adsorption of the charge in the electrolyte on the electrode surface, then the increase of current corresponds to the nucleation process, and the subsequent current stabilization stage is the growth of crystal nuclei. The cathode current for depositing ZnO stabilized at -1.4 mA cm^−2^, whereas the stabilized current was increased to −2.1 mA cm^−2^ with the addition of GO to the electrolyte for preparing ZnO-rGO composite, suggesting that GO promoted the growth rate. It is well-known that GO can be reduced under negative potential (Yang et al., 2014). Thus, the deoxidation of GO and the electrodeposition of ZnO should proceed concurrently. As a comparison, the deposition without adding LiClO_4_ as the supporting electrolyte, the current was steadied at −1.7 mA cm^−2^. This result suggests that the supporting electrolyte will increase the conductivity of the electrolyte, which is beneficial to the growth of the ZnO-rGO composite.

**FIGURE 2:**
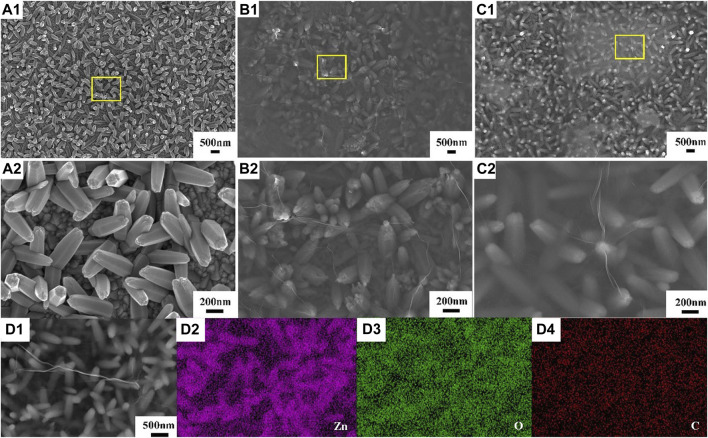
SEM micrographs of ZnO **(A)**, ZnO-rGO-N **(B)**, and ZnO-rGO **(C)** deposited on the FTO substrates, EDS elemental mapping of ZnO-rGO composite **(D)**.

Figure 2 displays the SEM micrographs of the ZnO, ZnO-rGO-N, and ZnO-rGO deposited on FTO substrates. As seen from Figure 2A, the pure ZnO has a uniform and dense hexagonal rod structure and completely covers the conductive substrate. As for ZnO-rGO-N deposited without supporting electrolyte, the morphology of the electrodeposited ZnO changed to a pencil shape with a larger size (Figure 2B), corresponding to a smaller specific surface area. In addition, rGO sheets were coated on ZnO nanorods due to the addition of GO. With the addition of LiClO_4_ as a supporting electrolyte, the diameter of ZnO nanorods in the ZnO-rGO composite material becomes smaller in diameter but larger in density (Figure 2C), leading to much larger surface area. The elemental mapping of ZnO-rGO displayed in Figure 2D proves the uniform distribution of C and O on the top of ZnO-rGO, suggesting the uniform coverage of rGO nanosheets on the top of ZnO nanorods.

**FIGURE 3:**
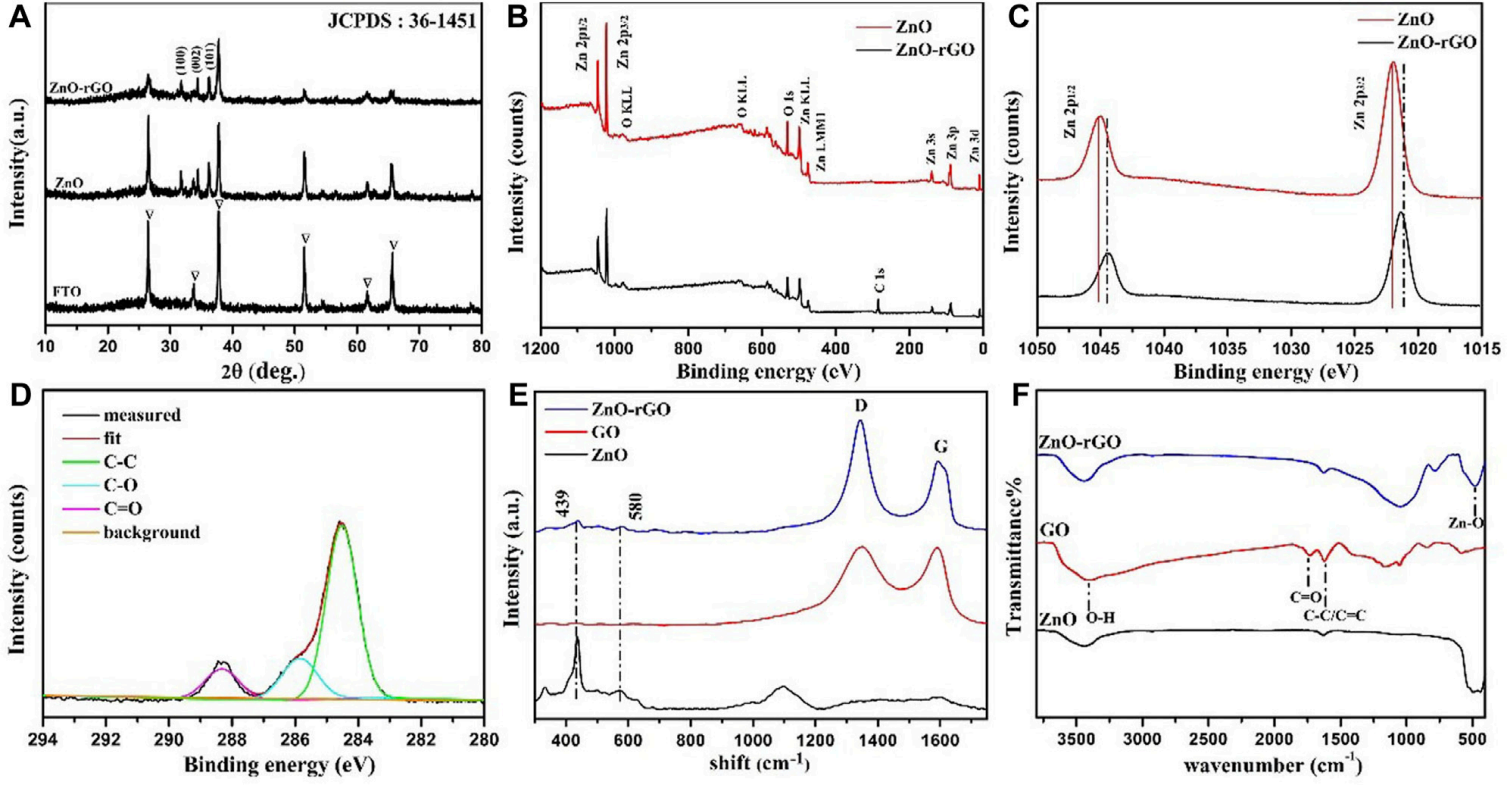
XRD patterns of ZnO and ZnO-rGO **(A)**; XPS survey spectra of ZnO and ZnO-rGO photoanodes **(B)**, Zn2p high-resolution spectra of ZnO and ZnO-rGO **(C)**, C1s scan spectrum of ZnO-rGO photoanode **(D)**; Raman spectra of ZnO-rGO and GO composite materials **(E)** and FTIR spectra of GO, ZnO and ZnO-rGO composites **(F)**.

Figure 3 shows the XRD patterns of ZnO and ZnO-rGO. In the pattern of ZnO, the high crystallinity ZnO with hexagonal wurtzite phase (JCPDS 361–451) was confirmed by the 31.78°, 34.4°, and 36.2° characteristic peaks except the peaks of FTO corresponding to the planes (100) (002) and (101) respectively. As for ZnO-rGO, the intensities of ZnO peaks decrease due to the coverage of rGO nanosheets. However, the diffraction peaks of rGO cannot be observed, which is probably because that the rGO nanosheets is too thin to be detected by XRD signal.

Figure 3B shows the XPS survey scan spectra of ZnO and ZnO-rGO composite material. The wide range analysis pattern of ZnO-rGO consist of C1s, O1s, and Zn2p. The XPS Zn2p spectra of the ZnO and ZnO-rGO are presented in Figure 3C. The binding energy positions of 1045.1 and 1021.9 eV accord with the two atomic states of Zn2p 1/2 and Zn2p 3/2, confirming the +2 oxidation states of Zn atoms. The binding energy of the two Zn2p peaks shifted slightly, which is due to the hybridization with GO, resulting in the change of the electronic band structure of ZnO. In Figure 3D the C1s scan spectrum of Zn-rGO can be deconvoluted into three peaks centered at the binding energies of 284.3, 286.4 and 288.8 eV, which can be assigned to the carbon atoms of C–C, C–O, and C=O bonds of rGO respectively. Compared with the spectrum of GO Supplementary Figure S1, the intensity of C-O and C=O peaks reduced, indicating that most oxygen-containing groups were removed upon reduction.

The Raman spectra of GO, ZnO and ZnO-rGO composite are shown in Figure 3E. In the spectrum of ZnO-rGO two typical bands corresponding to wurtzite-type ZnO were observed, in consistence with the spectrum of ZnO. The non-polar optical phonon E_2H_ mode can be revealed by a band at 440 cm^−1^, and the band at 580 cm^−1^ is ascribed to the existence of oxygen vacancies, zinc interstitials and defect complexes (Chaudhary et al., 2018; Erdogan et al., 2021). In the higher wavenumber range, the G (1591 cm^−1^) and D peaks (1340 cm^−1^) respectively corresponding to graphitic domains and lattice defects of rGO can be observed (Agarwal and Zetterlund, 2020). Compared with GO, the D to G band intensity ratio of ZnO-rGO increases, suggesting the decrease of average size of the sp^2^ carbon domains caused by the generation of more vacant lattice sites through the removal of carbon atom accompanied with the oxygenated groups removal.

Figure 3F shows the FTIR spectra of GO, ZnO, and ZnO-rGO composite materials. The wide and high-strength band at 3400 cm^−1^ is attributed to the O-H stretching vibration of water molecules adsorbed on the sample. The peak at 1630 cm^−1^ is ascribed to the C-C/C=C stretching of sp^2^ carbon domains. The band near 500 cm^−1^ is originated from the vibration absorption peak of the Zn-O bond. The stretching vibration absorption peak of the carbonyl group (C=O) in the carboxyl group (-COOH) of GO at 1738 cm^−1^ vanished in ZnO-rGO, proving that GO was reduced in this process (Lu et al., 2021).

**FIGURE 4:**
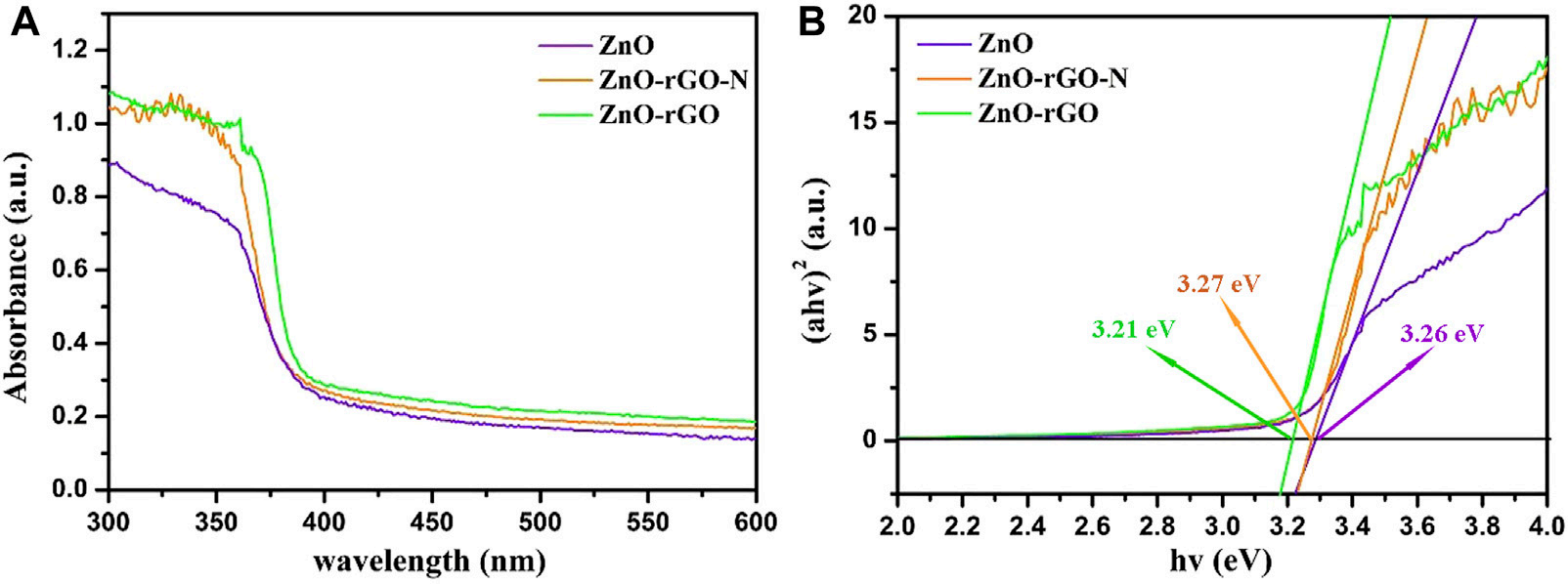
UV-vis absorption spectroscopy of ZnO, ZnO-rGO-N and ZnO-rGO composite materials **(A)** and the corresponding curves of (*αhv*)^2^ *versus* the *hv* **(B)**.

The UV-visible absorption spectra of ZnO and ZnO-rGO composite materials are described in Figure 4A. ZnO has obvious absorption in the UV region of 300–400 nm. As for ZnO-rGO-N, the absorbance in UV region increased slightly. In comparison, the absorbance of the ZnO-rGO composite materials enhanced significantly, especially in the area close to visible light (Vanitha et al., 2015). Besides, the absorption edge moves slightly towards the visible light region. The above outcomes indicate that more light can be absorbed for photocatalytic reaction due to the synergistic effect of rGO and ZnO.

According to the plots of (*αhυ*)^2^vs *hυ* displayed in Figure 4B, the bandgap can be calculated by the Kubelka-Munk method:

$$\alpha hv=A{\left(h\nu -E_{g}\right)}^{\eta }$$

where h , A , υ , and $E_{g}$ are Planck constant, absorption constant of direct transition, frequency of light, and the bandgap value, respectively. As we all know, *η* is an index that characterizes the light absorption process, and it is 1/2 for the of ZnO and ZnO-rGO composites with direct bandgap (Lupan et al., 2010). The calculated energy bandgap of ZnO is 3.29 eV. However, the bandgap of ZnO-rGO-N was reduced to 3.27 eV and further decreased to 3.21 eV for ZnO-rGO, which is beneficial to the absorption of photons with lower energy.

**FIGURE 5:**
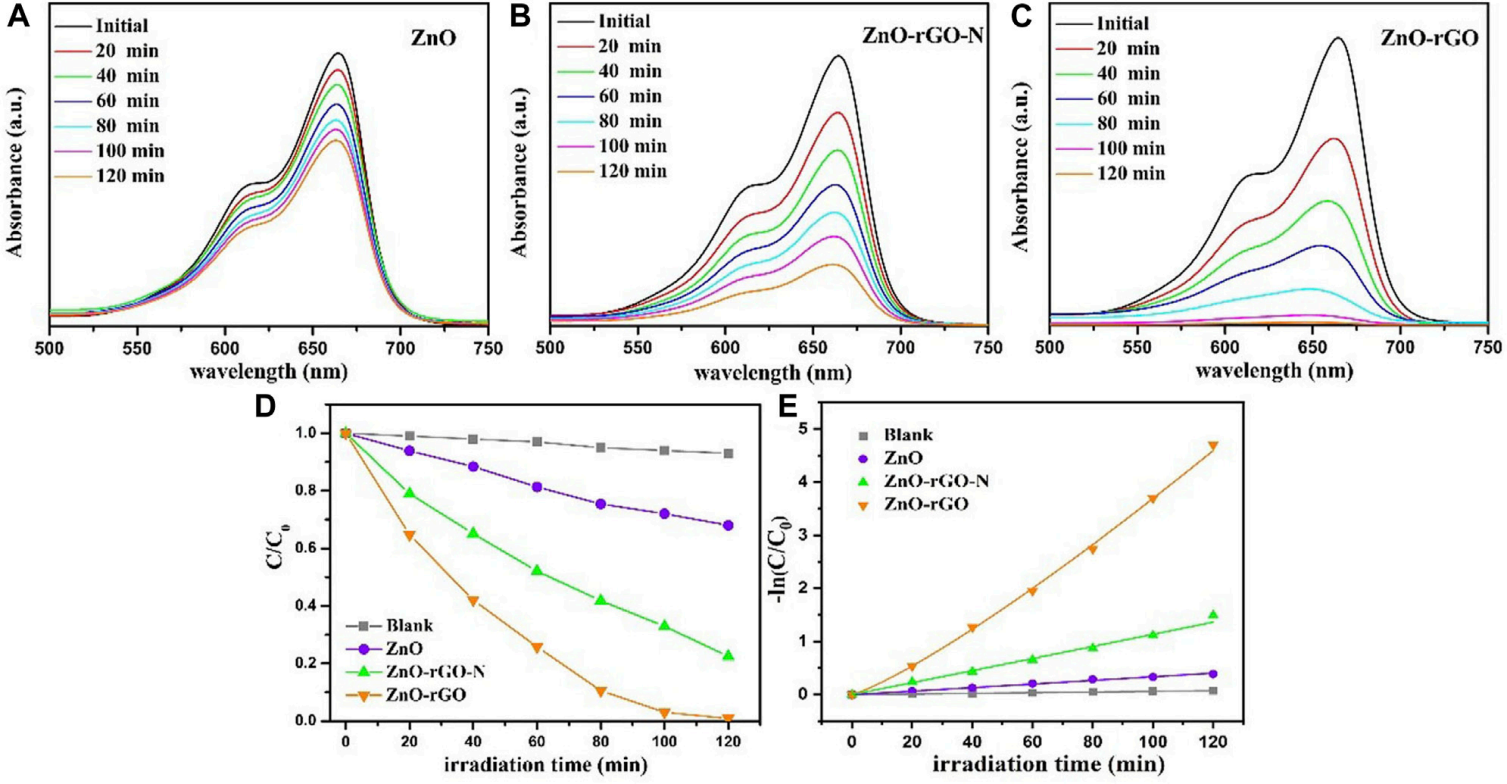
The changes of UV-vis spectra of dye degraded by ZnO **(A)**, ZnO-rGO-N **(B)** and ZnO-rGO **(C)** composites as interval illumination times, variation ratios of the concentrations of the MB degraded by different catalysts *versus* irradiation time **(D)**, and the corresponding kinetic curves **(E)**.

To assess the photocatalytic performance, ZnO, ZnO-rGO-N and ZnO-rGO composites were used to degrade methylene blue (MB) under mercury lamp irradiation. Figures 5A–C exhibits the variation of the UV-Vis absorption spectrum of the ZnO, ZnO-rGO-N and ZnO-rGO degraded MB solution with irradiation time. Compared with ZnO and ZnO-rGO-N, the absorption peaks of ZnO-rGO shrank rapidly with the largest extent as the extension of the irradiation time, and the absorption peak almost disappeared in 120 min. Figure 5D shows the variation of the MB concentrations by the degradation of different samples under a mercury lamp. The degradation rate can be calculated by Lambert-Beer law:

$$Degradation\;rate=\frac{C_{0}-C}{C_{0}}\times 100\%=\frac{A_{0}-A}{A_{0}}\times 100\%$$

where $C_{0}$ is the initial concentration of MB, C is the concentration of MB at time t, $A_{0}$ is the initial absorbance of MB solution, and A is the absorbance of MB solution at time t. As seen from Figure 5D, the MB degradation rate without using photocatalyst was only 7% under irradiation for 120 min, suggesting the MB can hardly be degraded without the aid of photocatalyst. As for ZnO catalyst, it can only degrade 32% of the MB dye within 120 min. After the incorporation of rGO nanosheets, the degradation rates of MB can reach about 77.5%, and 99.1% for MB dye in 120 min by the catalysis of ZnO-rGO-N and ZnO-rGO composites. This result suggests that the photocatalytic performance of ZnO can be significantly improved by the addition of rGO, probably be owing to that the presence of rGO reduced the recombination speed of electron-hole pairs and enhanced the absorption of MB (Kwon et al., 2017). Concurrently, the improvement of photocatalytic degradation efficiency of ZnO-rGO compared with ZnO-rGO-N should be attributed to the finer and more uniform ZnO nanorods (Figure 2), the increase of absorbance in visible region as well as the decrease of band gap width (Figures 4A,B).

According to the above results, the process of degradation of organic dyestuff by ZnO, ZnO-rGO-N and ZnO-rGO composite materials conformed to the first-order reaction kinetics:

$$-\frac{dC}{dt}=k\times C$$

$$-\ln\left(\frac{C}{C_{0}}\right)=k\times t$$

$$\ln\left(\frac{C_{0}}{C}\right)=kt$$

Here, k (min^−1^) represents the first-order rate constant, and t is the irradiation time. Figure 5E shows the first-order kinetic fitting results of these photodegradation, from which it can be distinctly found that the degradation rates follow the sequence: ZnO < ZnO-rGO-N < ZnO-rGO.

**FIGURE 6:**
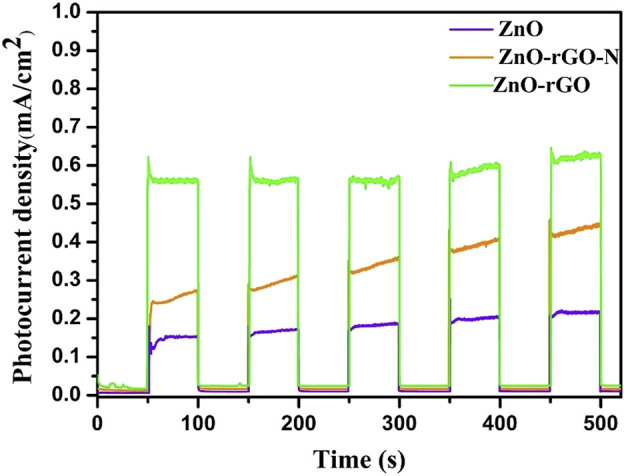
Transient photocurrent curves of ZnO, ZnO-rGO-N and ZnO-rGO.

The separation of photogenerated carriers during the photocatalytic process was demonstrated by the transient photocurrent curves of the electrochemically tested samples. In transient photocurrent curves, the carrier separation efficiency of the photocatalyst is proportional to the photocurrent corresponding. As can be seen in Figure 6, the photocurrent density of the photocatalyst in the absence of light is located at zero, and the photocurrent density rises rapidly after light exposure and stabilizes at a maximum point with good stability of the cycle within 500 s of discontinuous irradiation. The ZnO-rGO sample shows the maximum photocurrent stability density compared to ZnO and ZnO-rGO-N, which is due to the fact that the addition of rGO can effectively increase the conductivity, improve the electron transfer rate, and promote the separation of photogenerated electron-hole pairs.

**FIGURE 7:**
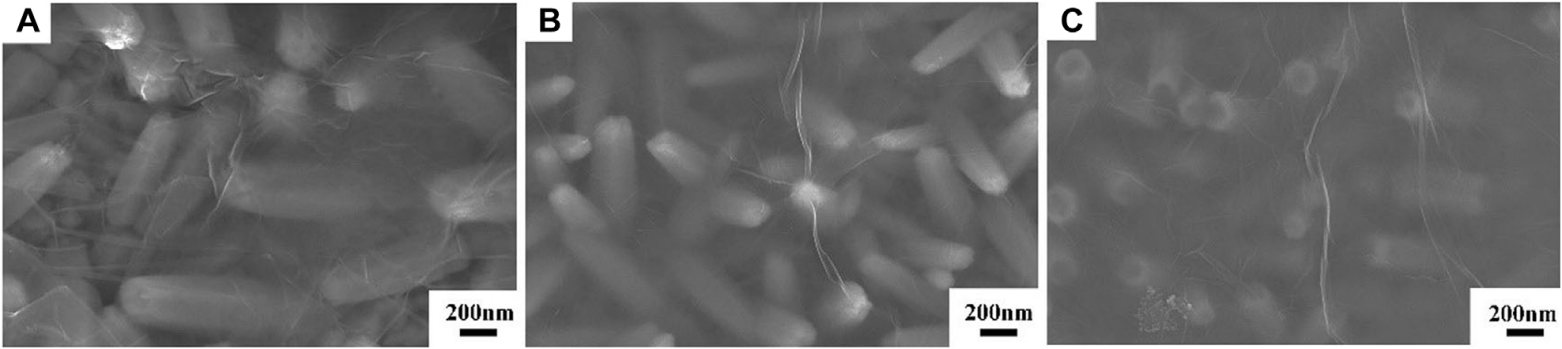
SEM micrographs ZnO-2rGO **(A)**, ZnO-5rGO **(B)**, and ZnO-8rGO **(C)**.

The morphologies of the ZnO-rGO composites prepared with electrolytes of different GO concentrations are depicted in Figure 7. In Figure 7A and 7B, the diameter of the ZnO nanorods reduced from 148.49 to 93.3 nm as the GO concentration was raised from 2 to 5 mg L^−1^. When the concentration was further raised to 8 mg L^−1^, the top of the ZnO nanorods was destroyed and the distribution of the nanorods became sparser, leading to the exposure of the substrate (Figure 7C).

**FIGURE 8:**
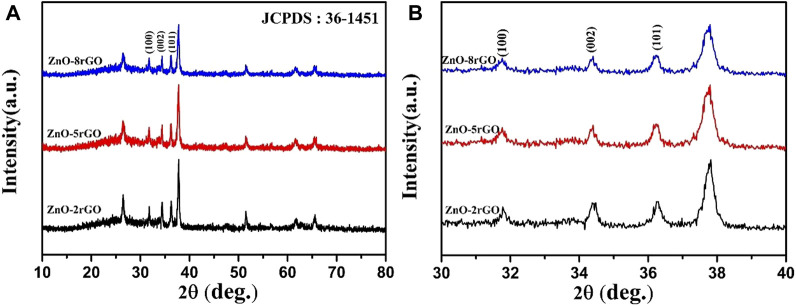
XRD spectra of ZnO-rGO composite prepared with different GO concentration **(A)** and partially enlarged pattern **(B)**.

Figure 8 exhibits the XRD spectra of pure ZnO and ZnO-rGO composites prepared with various GO concentrations. It can be seen that the characteristic peaks of ZnO and FTO decrease with the increase of GO concentration, which is because of the increased thickness of the rGO nanosheets covering the top of the ZnO nanorods in the ZnO-rGO composite material deposited on the FTO surface, in consistence with the results in Figure 7.

**FIGURE 9:**
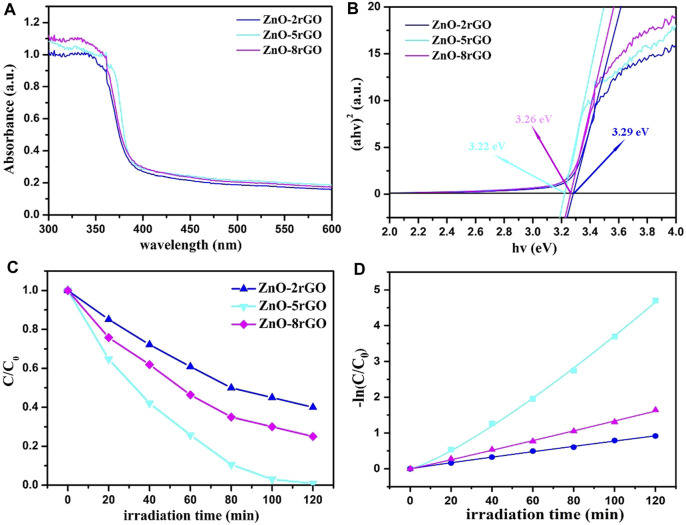
UV-vis absorption spectroscopy of ZnO-2rGO, ZnO-5rGO and ZnO-8rGO composite materials **(A)** and the corresponding curves of (*αhv*)^2^ *versus* the *hv* **(B)**; variation ratios of the concentrations of the MB degraded by different catalysts *versus* irradiation time **(C)**, and the corresponding kinetic curves **(D)**.

The UV-visible absorption spectra of ZnO-2rGO, ZnO-5rGO and ZnO-8rGO composite materials and corresponding curves of (αhv)^2^ *versus* the hv are described in Figures 9A,B. Compared with ZnO-2rGO, the absorbance of ZnO-5rGO and ZnO-8rGO increased significantly in the visible region, which was conducive to improving the photocatalytic degradation efficiency (Figure 9A). The calculated energy bandgaps of ZnO-2rGO, ZnO-5rGO and ZnO-8rGO from Figure 9B by the Kubelka-Munk formula are 3.29, 3.22, and 3.26 eV respectively.

Figure 9C shows the variation of the MB concentrations by the degradation of different samples under a mercury lamp, and the absorption spectra are shown in SupplementaryFigure S2. The degradation rates of MB dye by ZnO-2rGO, ZnO-5rGO and ZnO-8rGO composites can reach 60%, 99.1%, and 75% within 120 min. The ZnO-5rGO composites exhibit the best photocatalytic activity, which is ascribable to the largest surface area caused by the dense distribution of the ZnO nanorods with small diameter. The degradation rate of 99.1% for MB in 120 min is larger than previously reported ZnO-rGO composites, such as, ZnO/graphene composite prepared by surfactant-assisted simple hydrothermal method (90% in 130 min) (Zhou et al., 2012), ZnO-rGO composites fabricated by an easy one-step low-temperature chemical etching route (97% in 140 min) (Zhao et al., 2017), and ZnO/GO nanocomposite powder synthesized by novel flame transport approach (60% in 120 min) (Reimer et al., 2014).

**FIGURE 10:**
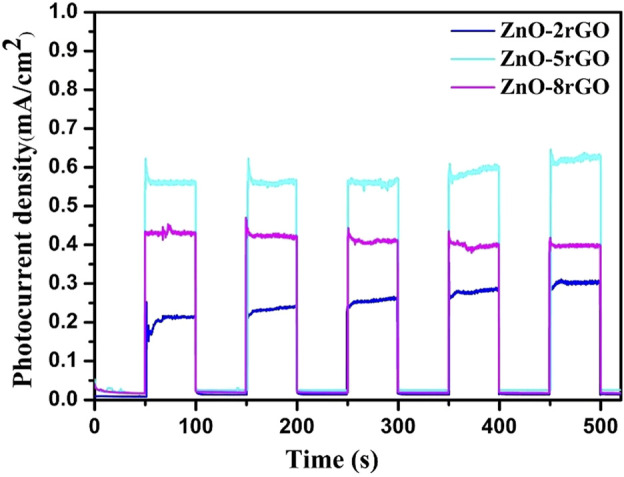
Transient photocurrent curves of ZnO-rGO prepared by different concentrations of precursors.

The photocurrent test can further illustrate the separation efficiency of photogenerated carriers during the reaction process. Figure 10 shows the transient photocurrent curves with ZnO-2rGO, ZnO-5rGO and ZnO-8rGO as photocatalysts. The degree of photocurrent response of ZnO-2rGO, ZnO-5rGO and ZnO-8rGO was different under 500 s intermittent irradiation. It indicates that the addition of different concentrations of GO in the precursor solution has an effect on the photocatalytic performance of the prepared ZnO-rGO samples, further verifying that the introduction of rGO with higher conductivity and larger specific surface area can be used as the acceptor and emitter of electrons to improve the conductivity and reduce the complexation of photogenerated carriers, resulting in improved photocatalytic performance. The photocurrent intensity of ZnO-5rGO is the largest compared with other samples, indicating that it has the best photocatalytic degradation performance.

**FIGURE 11:**
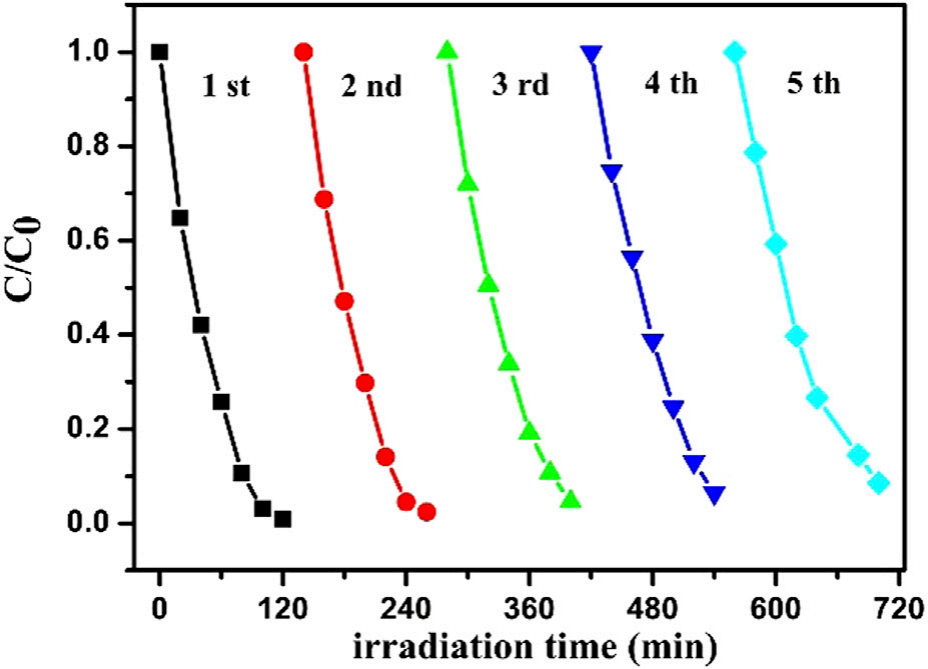
The photostability of ZnO-5rGO composite to MB degradation.

For further study the recyclability of ZnO-5rGO as a photocatalytic material, the degradation efficiency of repeatedly used ZnO-5rGO for MB was studied. After each cycle of degradation, the ZnO-5rGO coated FTO was washed with deionized water and then dried. As shown in Figure 11 and S3, the degradation efficiencies of the five cycles for MB are 99.1, 97.6, 95.4, 93.6, and 91.5%, respectively, suggesting the excellent reusability and light stability of the ZnO-rGO composite.

**FIGURE 12:**
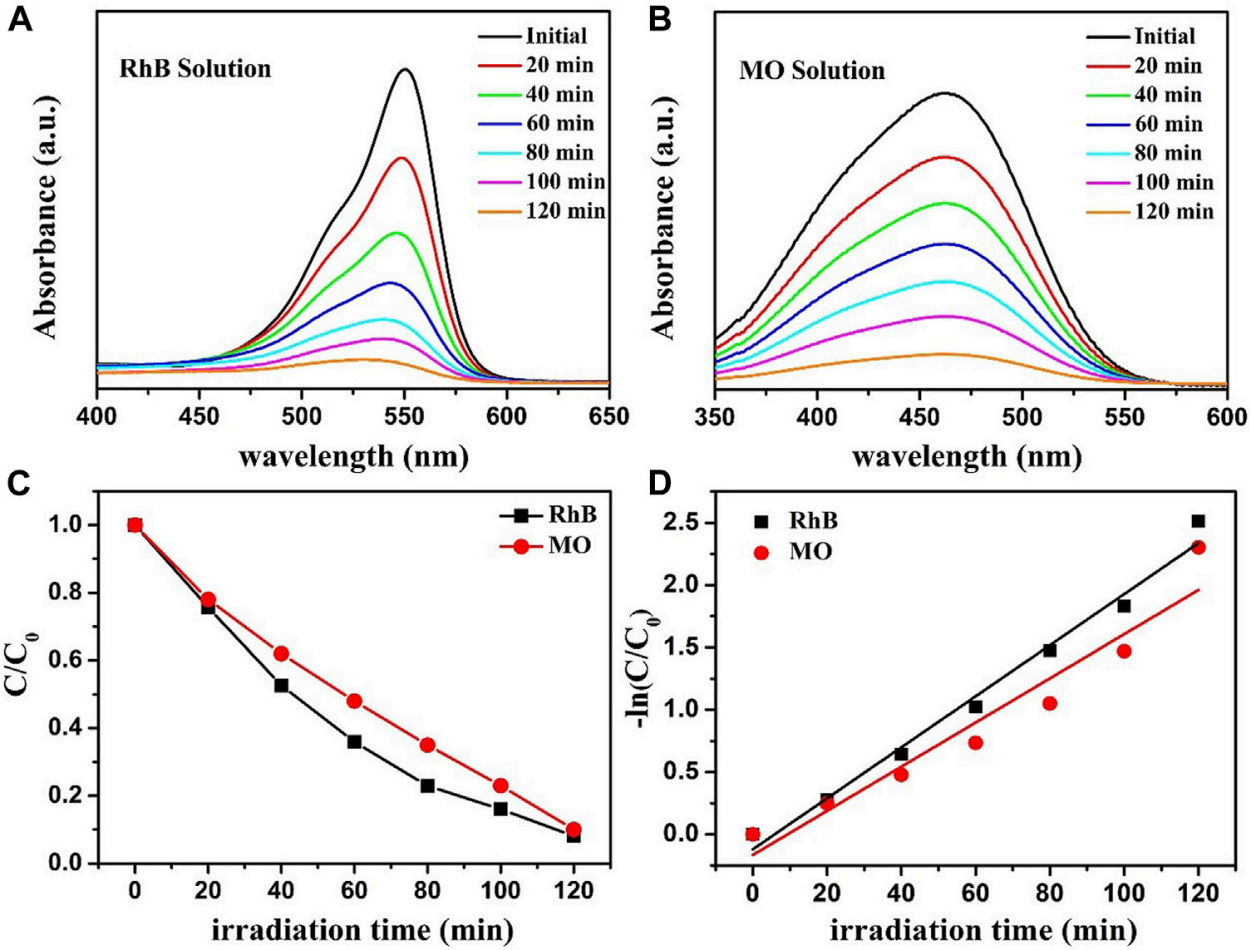
The absorption spectra of RhB **(A)** and MO **(B)** solutions that were photocatalytic degraded by ZnO-5rGO composites under mercury lamp irradiation; photocatalytic degradation of RhB and MO by the ZnO-5rGO composites **(C)** and the corresponding kinetic curves **(D)**.

To prove the universal application of ZnO-rGO composite for the degradation of dyestuffs, the degradation of RhB and MO solutions using ZnO-5rGO composites was studied, and the outcomes are presented in Figure 12. It can be observed from Figure 12A and 12B that absorption peaks of both RhB and MO decreased quickly under the irradiation of a mercury lamp. The photocatalytic degradation rate of MO and RhB solutions can be achieved to 90.3% and 93.2% in 120 min, respectively (Figure 12C). Combined with the first-order kinetic fitting results of photodegradation in Figure 12D, it can be concluded that ZnO-rGO composite also has excellent photocatalytic performance for other dyes.

**FIGURE 13:**
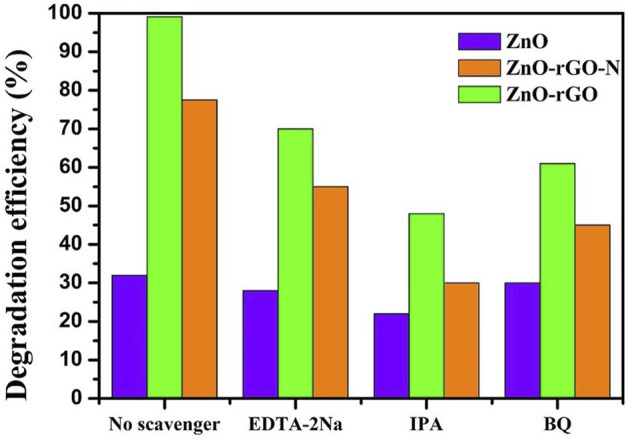
Degradation rate of MB degraded by ZnO, ZnO-rGO-N and ZnO-rGO Photocatalysts in the presence of capture agent.

To understand the main active species during the reaction of ZnO-rGO photocatalytic degradation of target degraded organics, the main active species during the photocatalytic reaction of ZnO, ZnO-rGO-N and ZnO-rGO photocatalysts for MB degradation without the addition of active species trapping agents and with EDTA-2Na, IPA and BQ as active species trapping agents are shown in Figure 13. The degradation rate of the target degradants after the addition of active species trapping agents was inversely proportional to the role played by the active species during the reaction. In Figure 13, the degradation rate of ZnO-rGO as a photocatalyst for MB was 99.1% without the addition of active species trapping agent, which changed to 70% with the addition of EDTA-2Na, 48% with the addition of IPA, and 61% with the addition of BQ. The changes in degradation rates after the addition of active species trapping agents indicate that OH is the active species that plays a major role in the photocatalytic degradation of MB.

**FIGURE 14:**
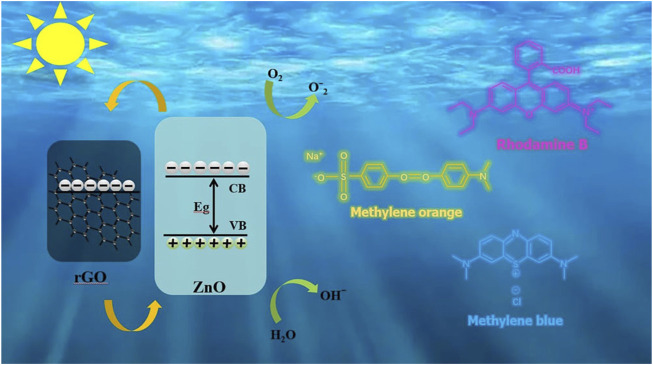
Diagram of the photocatalytic degradation mechanism of ZnO-rGO composites.

In this paper, there are many reasons why the photocatalytic degradation rate of ZnO-rGO composite was improved compared with that of ZnO. First, the ZnO nanorods of the synthesized ZnO-rGO composites are lower in diameter but higher in density compared with the pure ZnO, leading to a larger surface area. This will promote the spread and mass transfer of dye and oxygen species in photochemical reactions (Wei et al., 2013). Secondly, as shown in Figure 14, the coverage of rGO nanosheets on the top of ZnO nanorods can receive photo-generated electrons and accelerate charge separation through the charge transfer process. In addition, the generated charge can be quickly transferred, which is beneficial to the degradation of the dye (Kang et al., 2016).

## Conclusion

The ZnO-rGO photocatalyst was synthesized on the FTO substrate by a one-step electrochemical deposition method. After the incorporation of rGO into ZnO, the photocatalytic performance of the ZnO-rGO was significantly improved. The degradation of MB dye by ZnO-rGO composites synthesized with different concentrations of GO in electrolyte was also studied. The ZnO-rGO prepared with electrolyte containing 5 mg L^−1^ GO achieved the best photodegradation efficiency of 99.1% for degrading MB within 120 min. These outcomes indicate that the ZnO-rGO composites could be an excellent candidate material for photodegradation of organic dyes.

## Data Availability

The original contributions presented in the study are included in the article/supplementary material, further inquiries can be directed to the corresponding authors.
