# Supplementary material for: One-step electrodeposition of ZnO/graphene composites with enhanced capability for photocatalytic degradation of organic dyes
Source: Front Chem. 2022 Nov 2;10:1061129. doi: 10.3389/fchem.2022.1061129 (PMC9666895; doi:10.3389/fchem.2022.1061129)
Supplement: Supplementary file 1 [file DataSheet1.DOCX]

Supplementary Information

One-step electrodeposition of ZnO/graphene composites with enhanced capability for photocatalytic degradation of organic dyes

*Hui Lu ^12†^*, Simiao Sha^1†^, Tong Li ^1^, Qian Wen ^1^, Shaolin Yang ^1^ *, Jiandong Wu ^12^, Kang Wang ^a^, Zhilin Sheng ^a^, Jinfu Ma ^a^*

*^1^ School of Materials Science and Engineering,* *North Minzu University, Yinchuan 750021, China*

*^2^ Yinchuan Aini Industrial Technology Development Co., Ltd., Yinchuan 750021, China*

*Hui Lu ^12†^*and Simiao Sha^1†^ contributed equally to this work and share first authorship.*

*Corresponding authors. E-mail address:* [*luhui@nmu.edu.cn*](mailto:luhui@nmu.edu.cn) *(H. Lu)*

[*slyang@nun.edu.cn*](mailto:slyang@nun.edu.cn) *(S. Yang)*

**
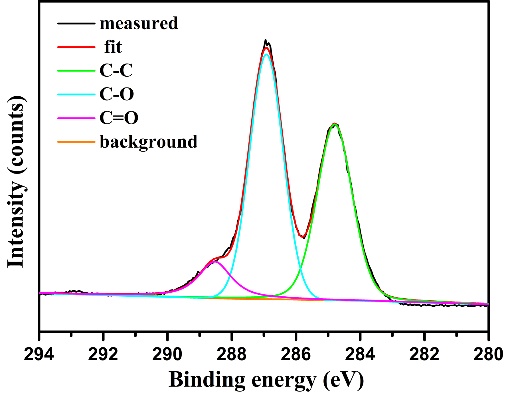
**

Figure S1. C1s XPS scan of graphene oxide.

**
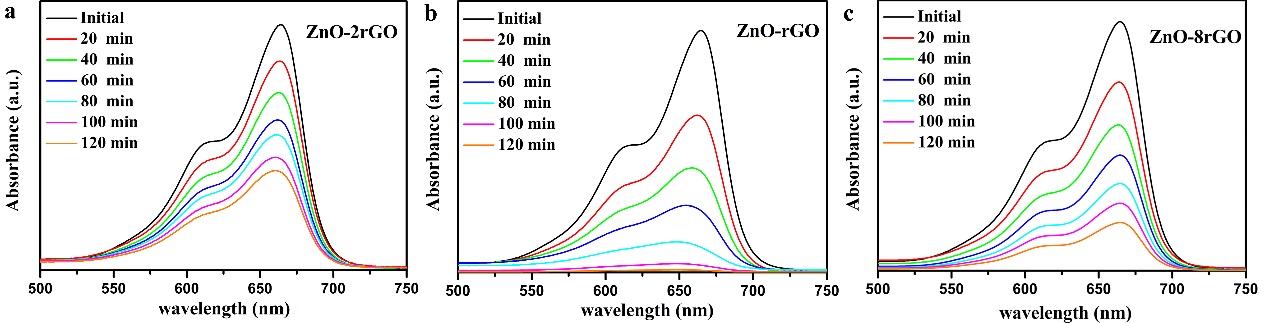
**

Figure S2. The changes of UV-vis spectra of MB degraded by ZnO-2rGO (a), ZnO-5rGO (b) and ZnO-8rGO (c) composites as interval illumination times.


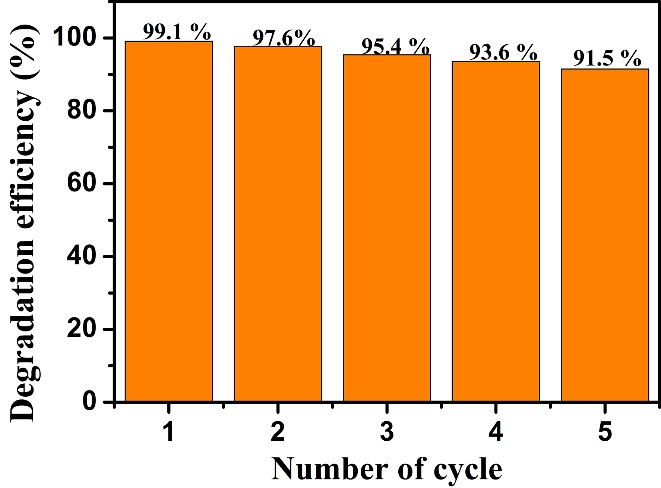


Figure S3. Histogram of photostability of MB degradation in ZnO-5rGO composites

over five cycles.
